# Effects of cardiac timing and peripheral resistance on measurement of pulse wave velocity for assessment of arterial stiffness

**DOI:** 10.1038/s41598-017-05807-x

**Published:** 2017-07-20

**Authors:** Hanguang Xiao, Mark Butlin, Isabella Tan, Alberto Avolio

**Affiliations:** 1 Chongqing Key Laboratory of Modern Photoelectric Detection Technology and Instrument, Chongqing University of Technology, No. 69 Hongguang Road, Banan District, Chongqing 400050 PR China; 2 Department of Biomedical Sciences, Faculty of Medicine and Health Sciences, Macquarie University, 2 Technology Place, Macquarie Park, NSW 2113 Australia

## Abstract

To investigate the effects of heart rate (HR), left ventricular ejection time (LVET) and wave reflection on arterial stiffness as assessed by pulse wave velocity (PWV), a pulse wave propagation simulation system (PWPSim) based on the transmission line model of the arterial tree was developed and was applied to investigate pulse wave propagation. HR, LVET, arterial elastic modulus and peripheral resistance were increased from 60 to 100 beats per minute (bpm), 0.1 to 0.45 seconds, 0.5 to 1.5 times and 0.5 to 1.5 times of the normal value, respectively. Carotid-femoral PWV (cfPWV) and brachial-ankle PWV (baPWV) were calculated by intersecting tangent method (cfPWV_tan_ and baPWV_tan_), maximum slope (cfPWV_max_ and baPWV_max_), and using the Moens-Korteweg equation (${\bf{cfPW}}{{\bf{V}}}_{{{\bf{c}}}_{{\bf{0}}}}$ and ${\bf{baPW}}{{\bf{V}}}_{{{\bf{c}}}_{{\bf{0}}}}$). Results showed cfPWV and baPWV increased significantly with arterial elastic modulus but did not increase with HR when using a constant elastic modulus. However there were significant LVET dependencies of cfPWV_tan_ and baPWV_tan_ (0.17 ± 0.13 and 0.17 ± 0.08 m/s per 50 ms), and low peripheral resistance dependencies of cfPWV_tan_, cfPWV_max_, baPWV_tan_ and baPWV_max_ (0.04 ± 0.01, 0.06 ± 0.04, 0.06 ± 0.03 and 0.09 ± 0.07 m/s per 10% peripheral resistance), respectively. This study demonstrated that LVET dominates the effect on calculated PWV compared to HR and peripheral resistance when arterial elastic modulus is constant.

## Introduction

Pulse wave velocity (PWV) has been considered an important indicator of arterial stiffness and a useful cardiovascular clinical marker^1, 2^. Although the effects of age, blood pressure and body height on PWV^3^ have been described and its standardization and reference values are being established^1^, there is still debate on the confounders of PWV assessment, such as heart rate (HR), left ventricular ejection time (LVET) and peripheral resistance^4–8^. Although some studies on animals and pacemaker patients found HR dependency of PWV^4, 9^, others claimed there was no effect of HR on PWV^10, 11^, and some concluded PWV was critically dependent on the method used to determine propagation time^12^. A recent study found that it was LVET, not HR, that independently correlated with aortic PWV^8^. Peripheral resistance is well known as a dominating parameter of the mean pressure in an arterial tree for a given input flow^13^. It also affects the magnitude and timing of wave reflection towards the heart^14^. However, the effect of peripheral resistance on PWV is still not clear. There are many complex interactions among the hemodynamic factors responsible for these controversial conclusions, thus making it difficult to investigate the independent effects of each factor on PWV.

A potential and feasible way to overcome this difficulty is to use numerical hemodynamic simulation of arterial pulse wave propagation. A transmission line model (TLM) of the human arterial system as a one-dimensional model is effective for simulation of pulse wave propagation^15–22^. TLM was proposed and further developed by previous investigators^22–27^. Based on their work, some researchers proposed electrical analogue models^20, 28, 29^, impedance models^19, 22, 30^ and multi-scale coupled models^17, 31, 32^. TLM has obvious advantages over these models in terms of less computational cost and more accurate description of pulse wave formation and development. It allows blood pressure and flow waveforms to be obtained at any point in the arterial tree and thus allows for analysis of the effects of various factors on pulse wave propagation. Although TLM has gained success in hemodynamic simulation, a lack of readily available simulation tools based on TLM presents a bottleneck to study the effects of confounders on PWV and other indexes.

In this study, we developed a pulse wave propagation simulation system (PWPSim) of the human arterial tree based on our previous studies of TLM^15, 16, 30, 33^. The effects of HR, LVET, arterial stiffness and peripheral resistance on carotid-femoral PWV (cfPWV) and brachial-ankle PWV (baPWV) were investigated by the simulation system. This study describes (i) a brief theory of TLM; (ii) solution strategy and calculation method of TLM; (iii) simulation of a standard human arterial tree using the PWPSim; (iv) study of the effects of HR, LVET, arterial stiffness and peripheral resistance on cfPWV and baPWV.

## Results

### Study 1: Comparison of PWV calculated by two timing methods and theoretical method under conditions of different arterial stiffness

**Figure 1:**
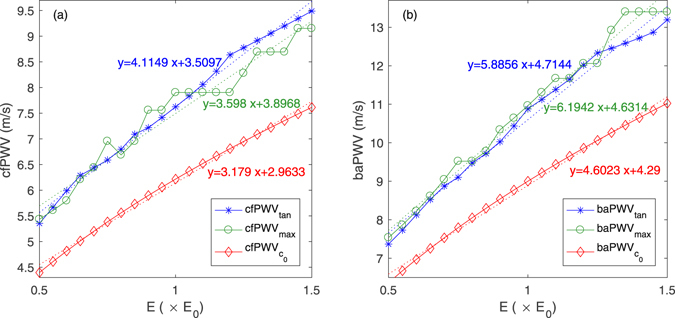
The effects of Young’s modulus on (**a**) cfPWVs and (**b**) baPWVs.

Figure 1 shows the effects of arterial elasticity described by Young’s modulus E on PWV. With increasing E, both cfPWV and baPWV increased gradually regardless of the calculation method of PWV. For cfPWV, cfPWV_tan_ by intersecting tangent method, cfPWV_max_ by the maximum slope method and ${{\rm{cfPWV}}}_{{{\rm{c}}}_{0}}$ by theoretical method increased by 4.1 m/s, 3.7 m/s and 3.2 m/s, respectively, as E increased from 50% to 150% E_0_. cfPWV_tan_ had a significant positive association to cfPWV_max_ and ${{\rm{cfPWV}}}_{{{\rm{c}}}_{0}}$ with a correlation coefficient 0.979 (P < 0.001) and 0.998 (P < 0.001), respectively. cfPWVtan was 0.1 ± 0.3 m/s higher than cfPWV_max_, and 1.5 ± 0.3 m/s higher than ${{\rm{cfPWV}}}_{{{\rm{c}}}_{0}}$. As for baPWV, baPWV_tan_, baPWV_max_ and ${{\rm{baPWV}}}_{{{\rm{c}}}_{0}}$ increased by 5.8 m/s, 5.9 m/s and 4.7 m/s, respectively, with increasing E. The mean difference between baPWV_tan_ and baPWV_max_ was −0.2 ± 0.2 m/s, and 1.7 ± 0.4 m/s between baPWV_tan_ and ${{\rm{baPWV}}}_{{{\rm{c}}}_{0}}$. baPWV_tan_ had a positive linear correlation to baPWV_max_ and ${{\rm{baPWV}}}_{{{\rm{c}}}_{0}}$ with a correlation coefficient of 0.993 (P < 0.001) and 0.998 (P < 0.001), respectively.

### Study 2: Effect of heart rate and LVET on PWV

**Figure 2:**
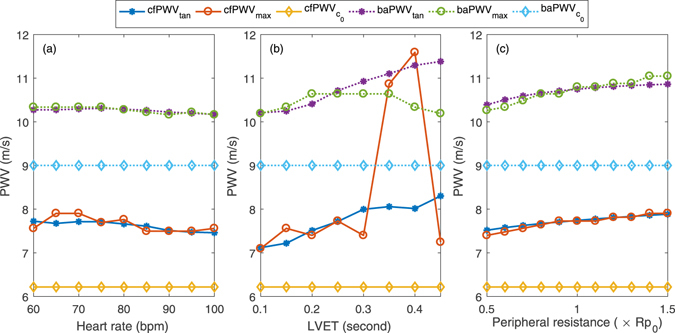
Effects of (**a**) heart rate, (**b**) LVET and (**c**) peripheral resistance on cfPWV and baPWV.

Figure 2(a,b) and Table 1 show the results of the effects of HR and LVET on cfPWV and baPWV. From Fig. 2(a), slight decrease in both cfPWV or baPWV was found with increasing HR. However, both cfPWV_tan_ and baPWV_tan_ increased significantly with LVET increasing from 0.1 to 0.45 seconds. There were no significant increases in cfPWV_max_ and baPWV_max_ as LVET increased, except the sharp increase after LVET 0.30 s.

**Table 1:** Correlation coefficients between PWV and HR and LVET, and their dependency of PWV (R represents correlation coefficient).

	R with HR	R with LVET	R with Rp	HR dependency of PWV (m/s/5 bpm)	LVET dependency of PWV (m/s/0.05 sec)	Rp dependency of PWV (m/s/10% Rp_0_)
cfPWV_tan_	−0.92**	0.97**	0.99**	−0.03 ± 0.04	0.17 ± 0.13	0.04 ± 0.01
cfPWV_max_	−0.60	0.51	0.97**	—	—	0.06 ± 0.04
baPWV_tan_	−0.80*	0.99**	0.95**	−0.01 ± 0.02	0.17 ± 0.08	0.06 ± 0.03
baPWV_max_	−0.91**	0.0	0.98**	−0.02 ± 0.04	—	0.09 ± 0.07

* and ** present P < 0.05 and P < 0.01, respectively.

Table 1 shows the correlation coefficients for cfPWV, baPWV, HR and LVET, as well their dependency effect on PWV. A slight negative dependency was observed between cfPWV or baPWV and HR except baPWV_max_. LVET was associated significantly with cfPWV_tan_ and baPWV_tan_ with correlation coefficients of 0.97 (P < 0.001) and 0.99 (P < 0.001), respectively. The corresponding LVET dependencies of cfPWV_tan_ and baPWV_tan_ were 0.17 ± 0.13 and 0.17 ± 0.08 m/s per 0.05 sec increase in LVET. The results indicated that LVET presented a greater influence on calculated cfPWV and baPWV than HR.

### Study 3: Effect of wave reflection on PWV

Figure 2(c) shows the results of the effect of peripheral resistance, as modelled by the combined resistance Rp of the two resistors R0 and R1 in the TLM, on terminal arteries on cfPWV and baPWV. There were obvious increases in both PWV_tan_ and PWV_max_. The correlation coefficients of cfPWV_tan_, cfPWV_max_, baPWV_tan_ and baPWV_max_ with the Rp were 0.99, 0.97, 0.95 and 0.98, respectively, all with P < 0.001. The peripheral resistance dependencies of cfPWV_tan_, cfPWV_max_, baPWV_tan_ and baPWV_max_ were 0.04 ± 0.01, 0.06 ± 0.04, 0.06 ± 0.03 and 0.09 ± 0.07 m/s per 0.1 Rp_0_ (default value), respectively. Although the correlation coefficients between the Rp and PWVs were high, the increases in the PWVs were very small with each 10% Rp_0_ increasing.

## Discussion

This study examined the individual effects of HR, LVET and wave reflection on calculated cfPWV and baPWV by the pulse wave propagation simulation system (PWPSim). It was found that, whilst HR did have a very slight effect on cfPWV or baPWV, increases in LVET and wave reflection arising from increased peripheral resistance resulted in higher calculated cfPWV and baPWV.

### Association between PWV and elastic modulus

Both cfPWV and baPWV computed by the intersecting tangent and maximum slope methods were higher than the theoretical values obtained with the Moens-Korteweg equation (Eq. 4), but all PWVs have significant positive association with the arterial elastic modulus. The main reason is that the Moens-Korteweg equation does not consider the dynamic elastic modulus and viscosity of arteries. Consequently, it would differ from the apparent PWV generated from arterial impedance^13^. Originally, cfPWV_tan_ and baPWV_tan_ were significantly higher than cfPWV_max_ and baPWV_max_ for each elastic modulus as the sampling frequency was high, which was consistent with the findings of previous studies^12^. This significant difference is reduced by increasing cfPWV_max_ and baPWV_max_ as decreasing the sampling frequency. However, the cfPWV_max_ and baPWV_max_ exhibit rapid change as the E and LVET are high, as shown in Figs 1 and 2, but cfPWV_tan_ and baPWV_tan_ show little change, which indicates the intersecting tangent method was more stable and robust than the maximum slope methods for the calculation of PWV and the estimation of arterial stiffness.

### Association between PWV and HR and LVET

Regarding the HR dependency of PWV, one potential reason for this may be due to arterial viscoelasticity. In this study, three constant values of elastic modulus were used for three major arterial types, and it was demonstrated that there was only a slight effect of HR on PWV. As to a positive HR dependency of PWV^6^, the frequency dependent elastic modulus E(*ω*) should be considered in future research. However, realistic E(*ω*) values for specific arteries are not available, even though some studies have addressed this field^34–37^.

**Figure 3:**
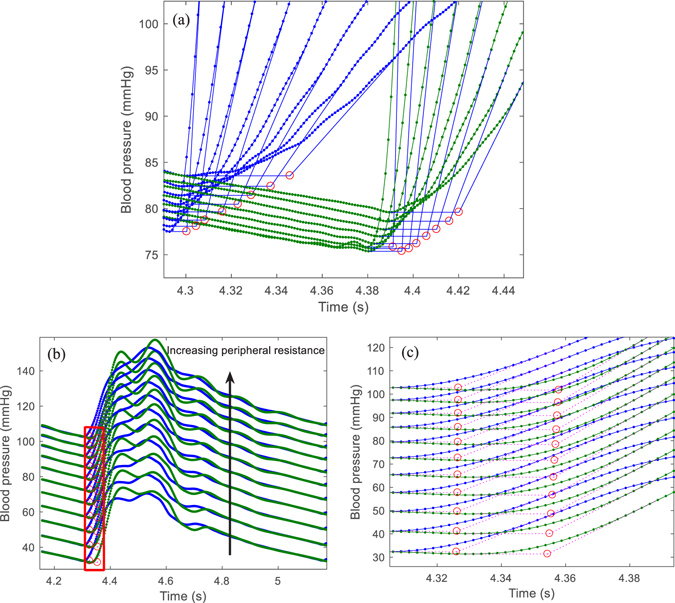
The schematic diagram of the changes of upstroke slopes and pulse foot in (**a**) the pulse waveforms of carotid (blue lines) and femoral (green lines) arteries with LVET and (**b**) the pulse waveforms of ascending aorta (blue lines) and carotid arteries (green lines) with peripheral resistance ((**c**) detail with enlarged scale for the red frame in left figure).

An interpretation of the positive correlation between PWV and LVET found in this study may be that the different LVET with a constant HR caused a change in the upstroke slope of pulse wave and so affected the calculation of transit time. This interpretation was supported by the simulation results as shown in Fig. 3(a), which shows the change of transit time with LVET between the carotid and femoral arteries. From Fig. 3(a), with increasing LVET, the upstroke slope of the blood pressure waveform of the carotid artery decreased, but at a faster rate than that of the femoral artery. Thus, the transit time decreased and so cfPWV increased. These results indicate that a sole change in LVET could not explain the negative association between PWV and LVET as found by other studies^8^; this would occur only if some structural or functional characteristics in the arterial wall changed with the increase in LVET.

### Association between wave reflection and PWV

Although cfPWV computed by using a foot-to-foot method to calculate pulse transit time is commonly considered to not be affected by wave reflection, slight increases in cfPWV and baPWV were found as peripheral resistance was increased in this study. The slight increase in cfPWV resulted from a non-changing ascending aortic-femoral PWV (afPWV) and a decreasing ascending aortic-carotid (acPWV) with increasing peripheral resistance. The effect of peripheral resistance on acPWV is shown in Fig. 3(b,c). The mean pressure and transit time, in Fig. 3(c), increased with increasing peripheral resistance. Increasing wave reflection resulted in an increased upstroke slope in the carotid artery, thus an increased transit time and a decreased acPWV. The same situation was also found in the brachial artery. Therefore, as generally expected, wave reflection has no significant influence on the foot of blood waveforms in central large arteries. However, it has a slight influence on those in peripheral arteries.

Load compliances (Cp) as another important parameter of terminal load also affects wave reflection but in a different way from peripheral resistance. Additional information would be better obtained by decoupling the steady and oscillatory effects in the arterial system, since wave reflections are essentially oscillatory phenomena and changing load compliances would not require further scaling of the inlet boundary flow to preserve the steady flow characteristics of the system (e.g. Mean Arterial Pressure, Total Peripheral Resistance)^38, 39^. However, the effect of load compliances on cfPWV_tan_ and baPWV_tan_ (−0.01 m/s and 0.02 per 10% Cp_0_) is less than that of peripheral resistance with change in all load compliances from 50% to 150% of normal reference value (Cp_0_) and simulated by the PWPSim system.

As for Rp, both resistors R1 and R0 of the 3-element Windkessel^40^ were altered simultaneously because both R1 and R0 contribute to the peripheral resistance of terminal arteries. In order to determine whether or not R0 has influence on PWV, both cfPWV and baPWV were calculated with increasing R0 whilst R1 was kept constant. The results showed that there was no change in either cfPWV or baPWV (data not shown), which agrees with the theoretical expectation that R0 only changes the DC impedance at 0 Hz, rather than the waveform and thus PWV. This means that the effect of peripheral resistance on PWV observed in this study was mainly attributed to R1. However, by definition, characteristic impedance of a lossless transmission line (a purely real number) should not dissipate energy. In addition, if all the terminal resistances were altered uniformly from half their default value to 1.5 times of their default value, perhaps the effect that peripheral resistance demonstrated on the carotid and brachial upstrokes in this study may be related to local disturbances induced by the impedance mismatch between the R1 and the characteristic impedance of artery linked to the R1. Since the carotid and brachial arteries are the smaller vessels studied here, they may be easily affected by mismatches between the terminal transmission line that connects to the lumped 3-element windkessel’s low resistance (high-frequency) resistor than the aorta and femoral. To further investigate this, the viscoelastic minimal model (VEMM)^41, 42^ may be more appropriate in studying the effect of peripheral resistance (DC impedance at 0 Hz). However, the values for all terminal arteries are not available for the VEMM, and thus it was not utilised in the presents study employing a multribranched arterial tree. Future studies are needed to determine whether there exists differences in the effect of wave reflection from the terminal arteries on PWV between the VEMM and the 3-element Windkessel model.

As to the TLM simulation, the PWPSim as a user-friendly tool not only provides a readily accessible means to investigate the effect of cardiovascular parameters on calaculated PWV, but also allows users to study other hemodynamic parameters for specific patients, such as central blood pressure, augmentation index, reflection amplitude, input impedance, characteristic impedance and transfer function. It can also be used to validate the theoretical feasibility of a new method or parameters for arterial functional diagnosis, such as arterial stiffness and stenosis before conducting a clinical tests^30, 43, 44^, and can be used to support the development and design of medical devices^45^. Some examples based on pulse wave simulation are illustrated by the following studies: Willemet *et al*. studied the accuracy of foot-to-foot pulse wave velocities for estimation of aortic stiffness by creating a database of 3,325 virtual healthy adult subjects using a validated one-dimensional model of the arterial hemodynamics^44^; Lillie *et al*. developed a nonlinear traveling wave model to analyze the impact of HR, blood pressure, and LVET on PWV^46^; Xiao *et al*. proposed and validated a novel method of artery stenosis diagnosis using transfer function and support vector machine based on TLM^30^; Vardoulis *et al*. established the relation of total arterial compliance and aortic PWV based on a hemodynamic simulation of 1000 virtual cases, which provided a new way to predict total arterial compliance by aortic PWV^47^.

In conclusion, TLM simulation showed that (1) HR has no significant association with PWV when constant values of elastic modulus of arteries are used; (2) LVET correlates positively with PWV when HR and the mean of flow are fixed; (3) peripheral resistance could slightly change PWV for central aorta, but the effect is only slight and so can be ignored to some extent.

## Methods

### Theory of transmission line model (TLM)

**Figure 4:**
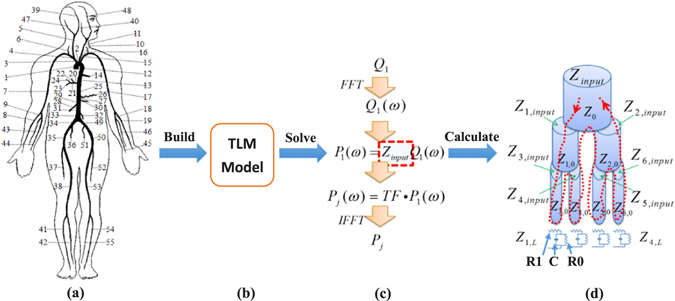
The schematic diagram of the model and solution: (**a**) the 55-segment model of human arterial tree, (**b**) TLM model, (**c**) solution strategy of the TLM model, and (**d**) calculation process of input impedance of a seven segment model.

TLM of the human systemic arterial tree was proposed based on a reduced arterial tree model consisting of main arteries, such as the 55-segment model, the 128-segment model or more complex models. The 55-segment model of human arterial tree is commonly used as shown in Fig. 4(a). This model also was used in this study based upon Stergiopulos’s version of data and modified according to recent studies, such as Westerhof *et al*.^48^, Avolio^22^, Stergiopulos *et al*.^49^, Wang and Parker^19^, Liang *et al*.^17^, and Alastruey and Parker *et al*.^50^. The TLM is based on the assumption that the human systemic arterial tree is a stationary and time invariant system, in which the formation of pressure waves follows the principle of superposition. Detailed vascular dimensions and elastic constants are described in our previous work^15^.

By definition, input impedance is the afterload of an arterial system presented at an arterial site, such as the No. 1 segment in Fig. 4(a). This is described by the ratio of sinusoidal functions of pressure and flow in the frequency domain. For an elastic arterial segment of length *l* and characteristic impedance *Z*_0_, the input impedance is defined as

1 $${Z}_{input}={Z}_{0}\frac{({Z}_{L}-{Z}_{0}){e}^{-\gamma l}+({Z}_{L}+{Z}_{0}){e}^{+\gamma l}}{({Z}_{0}-{Z}_{L}){e}^{-\gamma l}+({Z}_{L}+{Z}_{0}){e}^{+\gamma l}}$$

where *γ* and *Z*_*L*_ are the propagation constant and terminal impedance of the arterial segment. The characteristic impedance is

2 $${Z}_{0}=\frac{\rho {c}_{0}}{\pi {r}^{2}}{(1-{\sigma }^{2})}^{-\frac{1}{2}}{(1-{F}_{10})}^{-\frac{1}{2}}{e}^{j\varphi /2}$$

and propagation constant is

3 $$\gamma =\frac{j\omega }{{c}_{0}}{(1-{\sigma }^{2})}^{\frac{1}{2}}{(1-{F}_{10})}^{-\frac{1}{2}}{e}^{-j\varphi \mathrm{/2}}$$

where according to Womerley’s nomenclature, *F*_10_ = 2*J*_1_(*αj* ^3/2^)/(*αj* ^3/2^ *J*_0_(*αj* ^3/2^)), *J*_0_ and *J*_1_ are Bessel functions of order 0 and 1, respectively, and $j=\sqrt{-1}$; $\alpha =\sqrt{R\omega /\nu }$, and *ν* is the kinematic viscosity of blood; *ω* is the frequency; *σ* is the Poisson’s ratio of the artery wall; *c*_0_ is the pulse wave velocity defined by the Moens-Korteweg equation

4 $${c}_{0}=\sqrt{\frac{Eh}{\rho D}}$$

where *h* is the arterial wall thickness, *D* is the internal diameter, and *ρ* is blood density taken as 1.05 *g*/*cm* ^3^, *E* is the static Young’s modulus of the arterial wall.

There are two terminal types for an arterial segment: bifurcation or not. When the arterial segment is followed by a bifurcation which consists of two daughter arteries 1 and 2, the terminal impedance is

5 $${Z}_{L}=\frac{{Z}_{1,input}{Z}_{\mathrm{2,}input}}{{Z}_{\mathrm{1,}input}+{Z}_{\mathrm{2,}input}}$$

where *Z*_1,*input*_ and *Z*_2,*input*_ are the input impedances of two daughter arterial 1 and 2, respectively. When there is only one terminal arterial or a peripheral arterial, the terminal impedance is equal to the input impedance of the only daughter arterial or peripheral impedance.

The reflection coefficient at the terminal arteries can be defined as

6 $${\rm{\Gamma }}=\frac{{Z}_{L}-{Z}_{0}}{{Z}_{L}+{Z}_{0}}$$

According to transmission line theory, transfer function of an arterial segment *i* can be expressed as

7 $$T{F}_{i}=\frac{{P}_{distal}}{{P}_{proximal}}=\frac{1+{\rm{\Gamma }}}{{e}^{\gamma l}+{e}^{-\gamma l}}$$

If there is more than one artery connected in series between the two points of transfer function, the overall transfer function is obtained by multiplying all transfer functions together. For example, the transfer function between ascending aorta and tibial artery is calculated by

8 $$TF=\prod _{i=1,2,10,\ldots \mathrm{,31},\ldots \mathrm{,55}}\,T{F}_{i}$$

where *i* represents the number of artery between ascending aorta and tibial artery.

### Solution method of TLM

The main solution strategy of the TLM (Fig. 4(b)) is shown in Fig. 4(c). Firstly, blood flow *Q*_1_ of the ascending aorta was used as an input source to the arterial tree, and fast Fourier transforms of *Q*_1_ was performed to generate a flow spectrum *Q*_1_(*ω*). Then, *Q*_1_(*ω*) was multiplied by input impedance to produce a blood pressure spectrum *P*_1_(*ω*) at the inlet of the ascending aorta. Finally, multiplying *P*_1_(*ω*) with transfer function *TF*, then applying an inverse Fourier transforms $iFFT({P}_{1}(\omega )\times TF)$, the blood pressure waveform *P*_*j*_ of any one specific artery can be generated.

The aim, in this process, was to calculate input impedance of the ascending aorta. Automated calculation of input impedance is difficult as it is determined by the terminal resistances and compliances of all arteries in the arterial tree. However, a recursive algorithm was proposed in our previous work^15^ to implement the calculation of input impedance.

Figure 4(d) is an example of an arterial tree of seven arterial segments with four terminal impedances, which consists of a three-element Windkessel model to demonstrate the main idea of the recursive algorithm for calculating input impedance. Generally, according to the Eqs (1) and (4), if one wants to calculate *Z*_*input*_, *Z*_1,*input*_ and *Z*_2,*input*_ must first be calculated. In this way, all input impedance of the next generation of arteries, from *Z*_3,*input*_ to *Z*_6,*input*_, need to be calculated before *Z*_1,*input*_ and *Z*_2,*input*_. Therefore, the calculation of *Z*_*input*_ should begin at the terminal arteries and then back to the proximal arteries. However, for a complex arterial tree, it is difficult to calculate input impedance from generation to generation automatically. In order to calculate automatically, the recursive algorithm calculated input impedance from one side of arterial tree to another side, as indicated by the dotted line in Fig. 4(d). The computation sequence of parameters is ${Z}_{0}\to {Z}_{1,0}\to {Z}_{3,0}\to$ ${Z}_{1,L}\to {Z}_{\mathrm{3,}input}$ → ${Z}_{\mathrm{4,0}}\to \cdots \to {Z}_{\mathrm{6,}input}\to {Z}_{\mathrm{2,}input}\to {Z}_{input}$. The key of the recursive algorithm is to design a self-call subprogram to perform the calculation of input impedance for each unit consisting of characteristic impedance and one or more load impedance.

### Hemodynamic simulation for standard human arterial tree by the PWPSim

Based on the TLM model, solution strategy and calculation method, the pulse wave propagation simulation system (PWPSim) was developed by using MATLAB software (R2014a). The interface of the PWPSim is shown in the supplementary material. Its raw code can be freely downloaded from: https://www.researchgate.net/project/PWPSim-Pulse-Wave-Propagation-Simulation-System.

A base set of simulation parameters were used to model a standard human arterial tree, with an input source of heart rate, mean flow of the heart and LVET of 70 beats per minute (bpm), 70 ml/s and 0.3 second, respectively.

**Figure 5:**
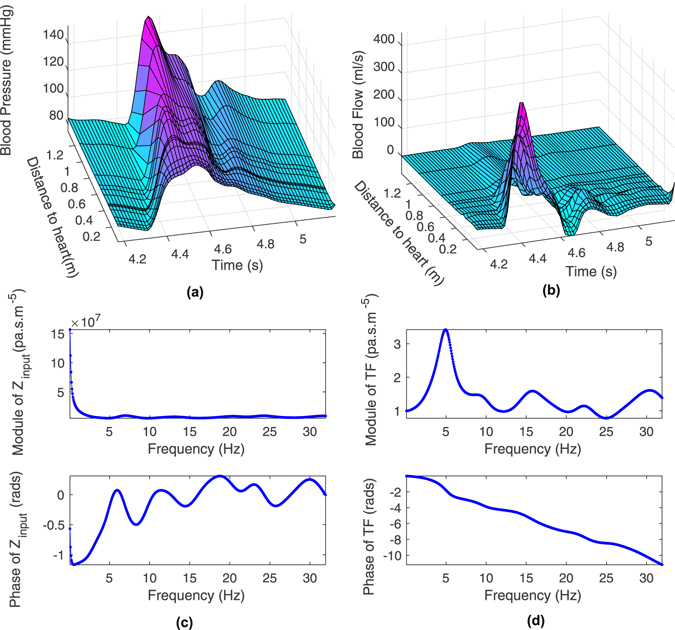
Simulation results: (**a**) blood pressure distribution and (**b**) blood flow distribution from aorta to tibial artery, (**c**) input impedance of ascending aorta and (**d**) transfer function between ascending aorta and brachial artery.

In order to observe the pulse wave propagation in the human arterial tree, the blood pressure and flow waveforms were calculated and displayed by the PWPSim using the default values representing the standard human arterial tree whose structural and functional parameters have been described in our previous work^15, 30^. The default Young’s modulus of the arteries has no frequency dependency. Figure 5(a,b) show the propagation of blood pressure and flow waveforms from aorta to tibial artery. In Fig. 5(a,b), the main characteristics of pulse wave propagation are displayed. For example, the time delay of wave propagation is evident from the foot of all waveforms. The systolic blood pressure increases gradually with increasing distance from the heart. The reflection wave in the peripheral artery is more obvious than in the proximal artery, which can be observed from the secondary hump after the end of systole caused by reflection from the vascular bed. In contrast, peak systolic flows decrease gradually along the arterial tree.

Input impedance of the ascending aorta of the standard arterial tree is shown in Fig. 5(c). From Fig. 5(c), one can observe quantitative information on the function of the arterial tree, such as total peripheral resistance (about $1.6\times {10}^{8}\,pa\cdot s\cdot {m}^{-5}$) and characteristic impedance (about $1.0\times {10}^{7}\,pa\cdot s\cdot {m}^{-5}$). Figure 5(d) also shows the simulation result of the transfer function between the ascending aorta and brachial artery. In Fig. 5(d), there is a peak in the modulus of the transfer function around 5 Hz. The modulus and phase of the TF also shows more oscillatory variation as frequency increases, which may be due to noise.

The detail blood pressure and flow values of six common arterial segments also are displayed in the table in the interface including ascending aorta, carotid artery, femoral artery, brachial artery, radial artery,and tibial artery. cfPWV and baPWV were calculated by dividing the length of the total sequence of arterial segments between carotid and femoral arteries or brachial and ankle arteries by the time delay extracted by the foot-to-foot tangential method, the maximum slope and Moens-Korteweg methods. For the base model of the human arterial tree used, simulation results of cfPWV and baPWV were 7.63 m/s and 10.90 m/s by the foot-to-foot tangential method. ABI is an indicator of arterial stenosis and is defined by dividing the SBP of the tibial artery and brachial artery. In the base model of the simulation, ABI was 0.89.

The blood pressure waveform can also be used to calculate the incident and reflected pressure wave by wave separation analysis (WSA) and thus reflected wave transit time and effective reflection distance^14^. For instance, a reflected wave transit time of 85 ms and an effective reflection distance of 32.5 cm was obtained by WSA from ascending aortic pressure waveform at a HR of 60 bpm and a LVET of 0.311 second.

### Simulation study on PWV

#### Study 1: Comparison of PWV calculated by two timing methods and theoretical method under different arterial stiffness

In order to investigate the effectiveness of the simulation system by the well-known relationship between arterial stiffness and PWV, the Young’s modulus of all arterial segments were changed from 50% to 150% of default value E_0_. Three fixed values of E_0_ were used, 4.0 × 10^5^ Pa for the large arteries, 8.0 × 10^5^ Pa for other aorta, 16.0 × 10^5^ Pa for peripheral arteries. A foot-to-foot intersection tangent method (corresponding to the subscript “tan”, e.g. PWV_tan_) and maximum slope method (corresponding to the subscript “max”, e.g. PWV_max_) were used to generate transit times from the ascending aorta to the femoral artery and from the brachial artery to tibial artery, respectively. The pulse path lengths of the four points (carotid, femoral, brachial and tibial arteries) from the heart were calculated as the total length of each arterial segment, and then subtracted the length of heart-to-femoral artery by that of heart-to-carotid artery to obtain the length of carotid-to-femoral artery, in the same way, the path length of brachial-to-tibial artery can also be obtained. Finally, cfPWV_tan_, cfPWV_max_, baPWV_tan_ and baPWV_max_ were calculated by dividing the path lengths by the corresponding transit times. At the same time, the theoretical values for ${{\rm{cfPWV}}}_{{{\rm{c}}}_{0}}$ and ${{\rm{baPWV}}}_{{{\rm{c}}}_{0}}$ were also calculated by the Morten-Kortesweg equation (Eq. (4)). The procedure of calculating ${{\rm{cfPWV}}}_{{{\rm{c}}}_{0}}$ included three steps: Firstly, PWV in each segment was calculated by Eq. (4). Secondly, the transit time of pulse in each segment was calculated by length/PWV. Finally, the difference of the sum of all segment lengths between the heart-femoral pathway and the heart-carotid was divided by the corresponding difference of the sum of all transit times to obtain the cfPWV. The calculation method of ${{\rm{baPWV}}}_{{{\rm{c}}}_{0}}$ is same to ${{\rm{cfPWV}}}_{{{\rm{c}}}_{0}}$.

#### Study 2: Effect of HR and LVET on PWV

In order to simulate the effects of HR on PWV, HR was specified to be 60 to 100 bpm with an interval of 10 bpm. The mean flow for each HR was normalized to a constant value of 70 ml/s to avoid an additional effect of mean flow on PWV. The cardiac period was calculated according to each HR. The LVET was calculated by a linear regression equation for a given HR^51^

9 $$LVET=-0.0017\times HR+0.413$$

**Figure 6:**
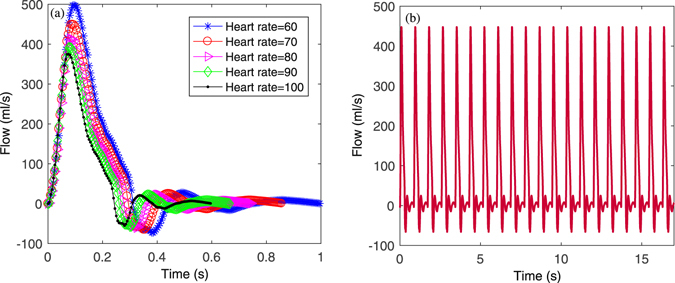
Input blood flow waveforms of model with (**a**) different heart rates and (**b**) a periodic flow waveform at heart rate 70 bpm fed to the TLM model.

The systolic and diastolic period of the typical flow waveform were scaled linearly to meet the constraints of LVET and cardiac period. Figure 6(a) shows the blood flow waveform at different HR. After periodic extension, these blood flows flow waveforms were (Fig. 6(b)) fed as input to the TLM.

As for the effect of LVET on PWV, in order to eliminate the effect of HR on LVET, a constant HR was used with different LVET values. HR was set to be 70 bpm. The mean of the input flow for each LVET was also normalized to 70 ml/s. LVET changed from 0.1 to 0.45 seconds in order to cover the main range of LVET^8^.

#### Study 3: Effect of wave reflection on PWV

Peripheral resistance from peripheral arteries directly affected pulse wave reflection. It is commonly thought that the calculation of PWV could not be influenced by the wave reflection caused by peripheral resistance^52^. Since both resistors (R1 and R0 in Fig. 4) of three-element Windkessel model contribute to the peripheral resistance of terminal artery, both resistors were changed at same time in our study. In order to validate whether or not the peripheral resistance affects PWV, the two resistors of three-element Windkessel model of all terminal arteries were changed from half of their default value (Rp_0_) to 1.5 times to study the effect of peripheral resistance on PWV. It should be noted that the peripheral compliance (Cp_0_) was kept constant.

### Statistical analysis

The correlation coefficients between each factors and cfPWV and baPWV were calculated and a P < 0.05 was considered statistically significant. Linear regression was used to describe the relation of the factors and cfPWV and baPWV. All data were presented as mean ± standard deviation. All statistical analysis were conducted in Matlab (R2014a) by using the built-in functions, such as ployfit, corrcoef, mean, std.

## Electronic supplementary material

Supplementary Information
