# Supplementary material for: Effects of cardiac timing and peripheral resistance on measurement of pulse wave velocity for assessment of arterial stiffness
Source: Sci Rep. 2017 Jul 20;7:5990. doi: 10.1038/s41598-017-05807-x (PMC5519778; doi:10.1038/s41598-017-05807-x)
Supplement: Supplementary file 1 — Supplementary Information [file 41598_2017_5807_MOESM1_ESM.pdf]

Effects of cardiac timing and peripheral resistance on measurement of pulse wave velocity for assessment of arterial stiffness

Hanguang Xiao[1,2], *Mark Butlin*[2], *Isabella Tan*[2], *and Alberto Avolio*[2,*]

Pulse wave propagation simulation system (PWPSim)

The interface of the pulse wave propagation simulation system (PWPSim) is shown as Figure S1. The system allows the user to specify different parameters of the human arterial tree and shows the simulation results as table, 2D or 3D figures and a complete report, and also allows the user to save all raw data for further analysis. The code of this simulation system can be freely downloaded from: https://www.researchgate.net/project/PWPSim-Pulse-Wave-Propagation-Simulation-System.

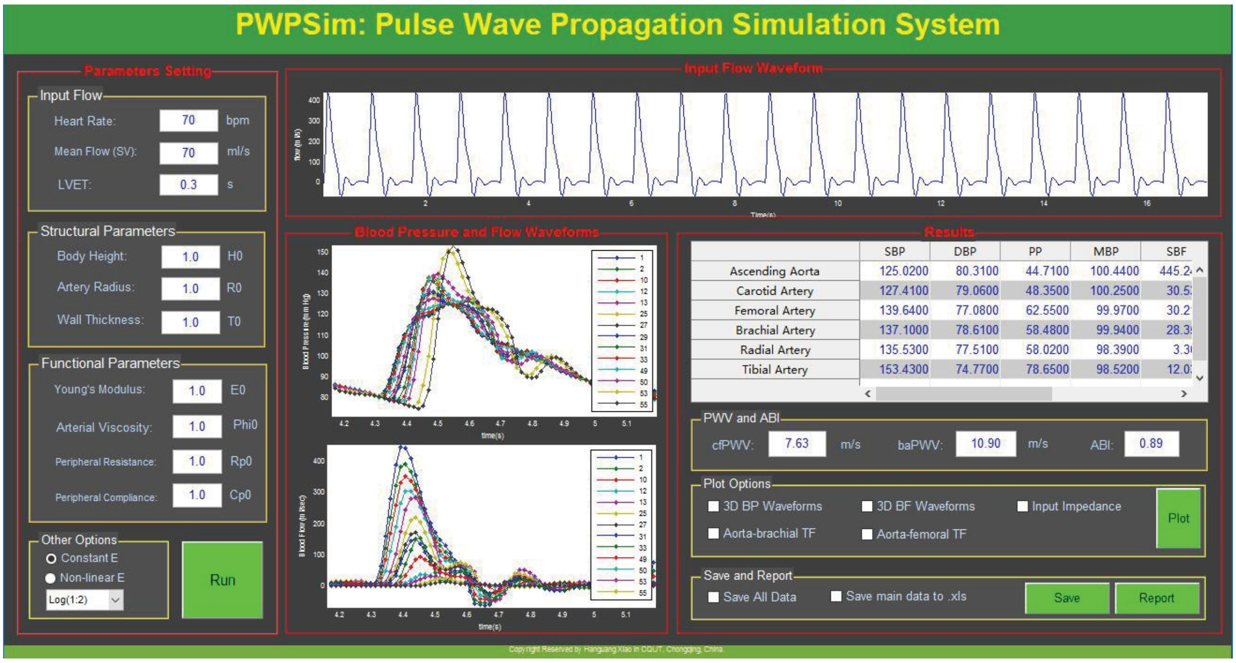

Figure S1. Pulse wave propagation simulation system (PWPSim)

It consists of four zones: Parameter Setting zone, Input Flow Waveform zone, Blood pressure and Flow Waveforms and Results zone. In Parameter Setting zone, four kinds of parameters can be modified according to user`s requirements, which include: (1) input flow parameters (heart rate (HR), mean flow and left ventricular ejection time (LVET)); (2) structural parameters (percent of standard body height, arterial radius and wall thickness); (3) functional parameters (percent of standard Young`s modulus, arterial viscosity, peripheral resistance and compliance); (4) special nonlinear Young`s modulus which depends on frequencies. After specifying a set of parameters, clicking Run bottom launches the calculation of TLM and the simulation results will display in one second.Input Flow Waveform zone will update the input flow according to the parameters set. The blood pressure and flow waveforms from ascending aorta to tibia artery were showed in the interface. In Results zone, the table shows some blood pressures and flow of the six interesting arteries, such as systolic blood pressure (SBP), diastolic blood pressure (DBP), pulse pressure (PP), and mean blood pressure (MBP). Blow the table, carotid-femoral

pulse wave velocity (cfPWV), brachial-ankle pulse wave velocity (baPWV) and ankle-brachial index (ABI) are shown and followed by Plot Options panel in which one can chose 3D blood pressure and flow waveforms, transfer function between aorta and femoral artery and input impedance of aorta to plot in each separate figure.
